# Physical Therapists’ Practices and Attitudes Toward Non-Steroidal Anti-Inflammatory Drugs: A National Cross-Sectional Study

**DOI:** 10.3390/healthcare14050591

**Published:** 2026-02-27

**Authors:** Samia A. Alamrani, Wadia S. Alruqayb, Hamad S. Al Amer, Sultan A. Alanazi

**Affiliations:** 1 Department of Health Rehabilitation Sciences, Faculty of Applied Medical Sciences, University of Tabuk, Tabuk 71491, Saudi Arabia; halamer@ut.edu.sa; 2 Department of Clinical Pharmacy, College of Pharmacy, Taif University, Taif 21944, Saudi Arabia; w.alruqayb@tu.edu.sa; 3 Department of Physical Therapy and Health Rehabilitation, College of Applied Medical Sciences, Majmaah University, Al-Majmaah 11952, Saudi Arabia; sa.alanazi@mu.edu.sa

**Keywords:** physical therapy practice, medication safety, musculoskeletal pain, Saudi Arabia

## Abstract

**Background:** Non-Steroidal Anti-Inflammatory Drugs (NSAIDs) are commonly used to manage acute or moderate-to-severe musculoskeletal pain. Physical Therapists (PTs) are involved in patient management from early on, providing education and advice related to medication use. This study aimed to examine Saudi PTs’ practice patterns and attitudes toward NSAID use and to identify factors associated with key practice outcomes by discussing NSAID use, assessing contraindications, monitoring side effects, and documenting discussions. **Methods:** A cross-sectional study was conducted between February and June 2025. A total of 371 PTs (52.3% male) from all regions of the country participated. Data were collected using an expert-reviewed and pilot-tested self-administered questionnaire. Descriptive statistics, chi-square tests, and logistic regression were used to analyze the data. Qualitative responses to an open-ended question were analyzed thematically. **Results:** Over half of PTs (59%) reported frequently discussing NSAID use with patients, particularly over-the-counter topical or oral formulations. Nearly half (48.0%) reported the absence of a formal institutional policy on NSAID discussions, while only 14.6% reported the presence of such policies. Safety practices were inconsistently applied: 46% reported screening for contraindications and 29% monitored potential long-term adverse effects (*p* < 0.001). Greater involvement in NSAID-related practices was associated with male gender, longer clinical experience, and specialist qualifications. Although 38% supported granting PTs hypothetical prescribing authority, 62% believed they lack adequate knowledge to provide safe and evidence-based medication advice. **Conclusions:** The study highlights the need for improved pharmacology education, clear national guidelines, and enhanced interprofessional collaboration to promote safe and consistent NSAID use in musculoskeletal care.

## 1. Introduction

Musculoskeletal conditions represent a major health problem, often leading to persistent pain, functional limitations, and long-term disability [1]. Physical Therapists (PTs), as primary practitioners in musculoskeletal rehabilitation, typically utilize non-pharmacological interventions such as therapeutic exercise, manual therapy and patient education as first-line management strategies [2]. However, acute flare-ups or moderate-to-severe musculoskeletal pain may necessitate the incorporation of pharmacological agents, including Non-Steroidal Anti-Inflammatory Drugs (NSAIDs) [3].

NSAIDs are widely used for their analgesic and anti-inflammatory properties [4]. They are also among the most commonly used medications alongside physical therapy interventions, whether prescribed or purchased over the counter [4]. Despite their therapeutic usefulness, NSAIDs are associated with dose- and duration-dependent risks, including gastrointestinal bleeding and ulceration, heightened cardiovascular complications, and renal impairments [5,6]. These potential risks highlight the importance of careful patient screening, accurate dosing, and awareness of drug interactions. Consequently, healthcare professionals involved in pain management must have detailed knowledge of NSAID contraindications and pharmacology, regardless of their prescribing authority.

In Saudi Arabia, the physiotherapy profession has grown considerably in recent decades, with increasing emphasis on evidence-based practice and multidisciplinary models of care [7]. Although PTs in Saudi Arabia cannot prescribe NSAIDs, their role as primary contact practitioners and patient educators places them in a strategic position to influence patients’ understanding, adherence, and safe use of these medications [8]. PTs are often responsible for integrating medication-related considerations into a patient’s active rehabilitation plan, identifying contraindications, and communicating medication-related concerns with prescribing physicians or pharmacists [9]. Research from international settings indicates that a significant number of patients undergoing physical therapy are concurrently taking NSAIDs [10,11], yet studies consistently highlight considerable variability and occasional gaps in PTs’ knowledge and confidence regarding pharmacological pain management [12,13,14]. Such variations underscore the need for context-specific evaluations.

Despite the critical role of PTs in the multidisciplinary pain management, there is a lack of data concerning their clinical practices and professional attitudes towards NSAIDs in the Kingdom of Saudi Arabia. Understanding such aspects is essential to improving patient safety, informing professional development and curriculum planning, and strengthening interprofessional care strategies [15]. Therefore, the primary objective of this study was to examine the clinical practices and professional attitudes of PTs practicing in Saudi Arabia regarding NSAID use in the management of musculoskeletal conditions. The secondary objective was to identify factors associated with their involvement in NSAID-related clinical practices. Specifically, this study sought to answer the following research questions: What are the current practice patterns and attitudes of Saudi PTs toward NSAID use, and which demographic or professional factors are associated with their involvement in NSAID-related clinical practices?

## 2. Materials and Methods

This study was conducted and reported in accordance with the Strengthening the Reporting of Observational Studies in Epidemiology (STROBE) recommendations [16] (Supplementary File S1). A cross-sectional study design was employed, using an online, self-administered questionnaire to assess PTs’ practices and attitudes toward NSAIDs. The survey was distributed to certified PTs across Saudi Arabia between February and June 2025.

### 2.1. Participants

Certified PTs currently living and working in Saudi Arabia across all settings were invited to participate. Consent was obtained electronically through the survey platform (Google Forms). Sample size was calculated using OpenEpi software (Version 3.01) [17], assuming a 95% confidence interval, 50% expected proportion, and 5% margin of error, yielding a target of 365 participants.

Inclusion criteria: Licensed male and female PTs practicing in Saudi Arabia who provided informed consent.

Exclusion criteria: Interns, physiotherapy assistants/technicians, retired professionals, and individuals who did not complete the questionnaire.

### 2.2. Measures

#### Questionnaire

The questionnaire was developed following a review of the relevant literature [12,18,19]. Initial items were generated based on previously published instruments and concepts relevant to NSAID-related practices and attitudes among healthcare professionals. To establish face and content validity, the draft questionnaire was reviewed by an expert panel consisting of three senior physical therapy academics with 10–18 years of clinical and research experience and one clinical pharmacist with 14 years of clinical and research experience. The panel qualitatively evaluated the questionnaire for clarity, relevance, and comprehensiveness and alignment with study objectives. Feedback was obtained through iterative discussion, and items were revised based on consensus recommendations. No formal quantitative content validity index was calculated. Prior to data collection, a pilot study was conducted with 10 practicing PTs to assess clarity, readability, and applicability. Minor modifications were made accordingly (Supplementary File S2). Data from the pilot study were not included in the final analysis.

The final questionnaire comprised four sections: consent and study information, demographics (11 items including age, sex, region, years of experience, and specialization), practice patterns (11 items assessing discussion of NSAID use, adherence to policy, patient assessment, and monitoring) and attitudes (4 items, including 3 closed and 1 open-ended question). For the purposes of this study, the term “discuss” referred to medication-related conversations conducted within existing scope-of-practice boundaries, whereas “prescribing” referred solely to participants’ attitudes toward hypothetical prescribing authority.

Formal psychometric reliability testing (e.g., internal consistency using Cronbach’s alpha or test–retest reliability) was not conducted, as the questionnaire primarily consisted of descriptive and standalone items rather than multi-item scales measuring latent constructs.

### 2.3. Data Collection

Ethical approval was obtained from the Ethics Committee at Taif University, Saudi Arabia (46-155). All participants provided informed consent prior to their inclusion in the study. Participation was voluntary, and respondents were informed of their right to withdraw at any time without any consequences. To ensure confidentiality, all data were collected anonymously, and no identifiable personal information was recorded. The questionnaire was hosted on Google Forms and distributed via social media (X, Telegram, and WhatsApp), emails, PT forums and professional associations using a snowball sampling approach. Data were collected anonymously and cleaned prior to analysis.

### 2.4. Statistical Analysis

Data were analyzed using SPSS version 26.0 (IBM Corp., Armonk, NY, USA). Descriptive statistics summarized participant characteristics, practice patterns, and attitudes. Differences across categorical variables were assessed using chi-square tests (*p* < 0.05 considered significant).

Binary logistic regression was conducted to identify factors associated with key practice outcomes (discussing NSAID use, assessing contraindications, monitoring side effects, and recording discussions in clinical records). Both univariate models (crude odds ratios [COR] with 95% confidence intervals [CI]) and multivariate models (adjusted odds ratios [AOR] with 95% CI) were used. Variables with *p* < 0.05 were considered statistically significant factors.

Multicollinearity was assessed using variance inflation factor (VIF), with a threshold of ≤5 indicating acceptable levels [20]. The VIF values ranged from 1.01 to 1.70 and were well below the cutoff of 5, suggesting the absence of multicollinearity.

Open-ended responses were analyzed thematically and categorized as supportive, opposing, or neutral/uncertain, with illustrative quotations included in the results.

## 3. Results

### 3.1. Participant Characteristics

A total of 371 PTs participated in the study; their mean age was 33.3 years (SD = 6.67). Slightly more than half were males (52.3%), while females represented 47.7%. Participants represented all regions of Saudi Arabia and varied in terms of gender, employment sector, level of experience, academic qualifications, and clinical specializations. The Western region contributed the largest proportion of respondents. Most participants held a bachelor’s degree and worked in public or private clinical settings, with orthopedics and neurology being the most common specialties. Detailed characteristics are presented in Table 1.

### 3.2. Practice Characteristics Regarding NSAID Use

A significant proportion (n = 178; 48.0%) reported that their institution had no formal policy on NSAID-related discussions, compared with only 14.6% (n = 54) who reported the presence of a policy (*χ*^2^ = 65.02; *p* < 0.001). Among those with a policy, almost all (n = 51; 94.4%) stated that they follow it (*χ*^2^ = 42.67; *p* < 0.001). Of those without a policy or who were unsure, more than two thirds (n = 217; 68.5%) believed that such a policy would be beneficial (*χ*^2^ = 178.39; *p* < 0.001).

**Figure 1:**
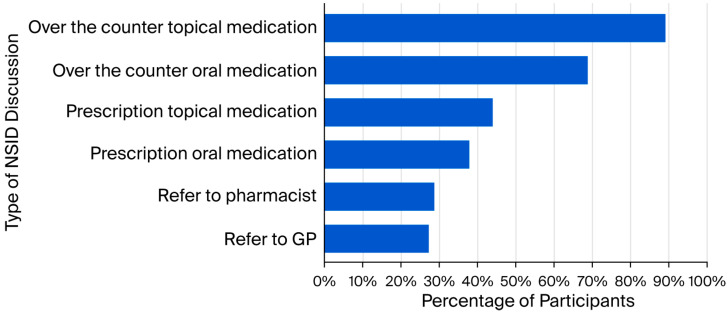
Types of NSAID discussions by physical therapists (n = 219; multiple responses).

More than half of participants (n = 219; 59.0%) frequently discussed NSAID use with their patients, compared with 41.0% (n = 152) who did not (*χ*^2^ = 12.10; *p* < 0.001). As depicted in Figure 1, this discussion most often involved over-the-counter topical (n = 193; 88.1%) and oral medications (n = 149; 68.0%), followed by prescription topical (n = 95; 43.4%) and oral medications (n = 82; 37.4%), pharmacist referral (n = 62; 28.3%), and general practitioner referral (n = 59; 26.9%).

**Figure 2:**
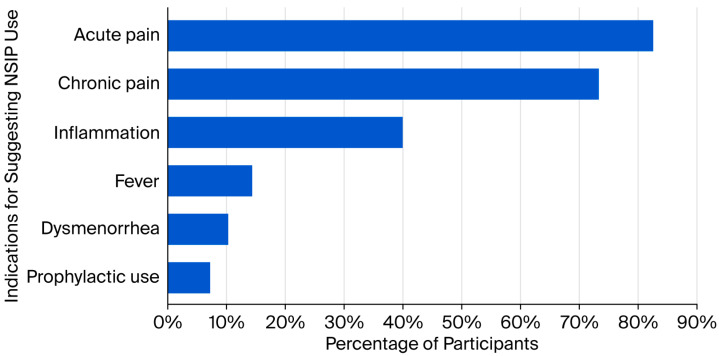
Common indications for suggesting NSAID use in physical therapy practice (n = 195; multiple responses).

Nearly half (n = 176; 47.4%) of PTs indicated that they never suggest NSAID use, while 26.1% (n = 97) reported doing so daily (*χ*^2^ = 228.8; *p* < 0.001). Smaller proportions reported suggesting NSAID use rarely (13.7%, n = 51), weekly (10.2%, n = 38), and monthly (2.4%, n = 9). The distribution was statistically significant (χ^2^ = 228.88, *p* < 0.001). As shown in Figure 2, suggestions for use were most commonly acute pain (n = 161; 82.6%) and chronic pain (n = 143; 73.3%), with fewer citing inflammation (n = 78; 40.0%), fever (n = 28; 14.4%), dysmenorrhea (n = 20; 10.3%), or prophylactic use (n = 14; 7.2%).

**Figure 3:**
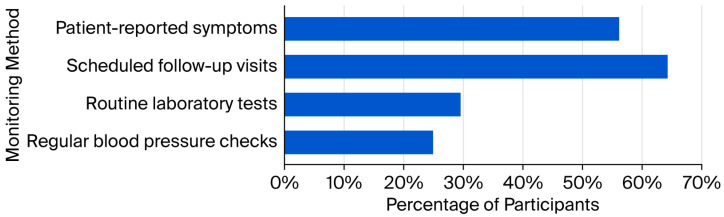
Monitoring patients on long-term NSAIDs for side effects (n = 109; multiple responses).

No significant difference was found between the proportion of participants who assessed contraindications before suggesting NSAID use (n = 172; 46.4%) and those who did not (n = 199; 53.6%) (*χ*^2^ = 1.97; *p* = 0.161). Only 29.4% (n = 109) monitored patients on long-term NSAIDs for side effects, compared with 70.6% (n = 262) who did not (*χ*^2^ = 63.10; *p* < 0.001). As shown in Figure 3, monitoring strategies included follow-up visits (n = 70; 64.2%), patient-reported symptoms (n = 61; 56.0%), laboratory tests (n = 32; 29.4%), and blood pressure checks (n = 27; 24.8%).

Finally, less than one third (n = 111; 29.9%) recorded discussions on medications in their clinical records, compared with 70.1% (n = 260) who did not (*χ*^2^ = 59.84; *p* < 0.001).

#### Scope of Discussing NSAID Use

Most PTs reported including indications when discussing NSAID use with their patients (n = 273; 73.6% vs. n = 98; 26.4%) (*χ*^2^ = 82.55; *p* < 0.001). In contrast, a substantial proportion did not address drug interactions (n = 240; 64.7%) compared with those who did (n = 131; 35.3%) (*χ*^2^ = 82.55; *p* < 0.001).

There was no significant difference between those who provided and did not provide warnings/advice (n = 203; 54.7% vs. n = 168; 45.3%) (*χ*^2^ = 3.30; *p* = 0.069), precautions (n = 179; 48.2% vs. n = 192; 51.8%) (*χ*^2^ = 0.46; *p* = 0.500), contraindications (n = 170; 45.8% vs. n = 201; 54.2%) (*χ*^2^ = 2.59; *p* = 0.108), side effects (n = 172; 46.4% vs. n = 199; 53.6%) (*χ*^2^ = 1.97; *p* = 0.161), or dosage information (n = 185; 49.9% vs. n = 186; 50.1%) (*χ*^2^ = 0.003; *p* = 0.959).

### 3.3. Associations Between Participant Characteristics and NSAID-Related Practices

#### 3.3.1. Discussing NSAID Use with Patients

In the univariate analysis (Table 2), male PTs were more likely than females to discuss NSAID use with patients (COR = 2.01, 95% CI: 1.32–3.06). Longer experience (1–5 years: COR = 2.64, 95% CI: 1.24–5.62; 6–10 years: COR = 2.82, 95% CI: 1.28–6.20; ≥11 years: COR = 3.69, 95% CI: 1.67–8.14), postgraduate degrees (COR = 5.45, 95% CI: 1.05–28.32), and orthopedic (COR = 2.04, 95% CI: 1.09–3.81), neurological (COR = 2.44, 95% CI: 1.21–4.89), and sports (COR = 2.40, 95% CI: 1.08–5.35) specializations were also significant predictors. In the multivariate model, male therapists (AOR = 1.72, 95% CI: 1.09–2.73) and those with 1–5 years (AOR = 2.44, 95% CI: 1.04–5.60) and ≥11 years of experience (AOR = 3.77, 95% CI: 1.31–10.86) remained significantly more likely to discuss NSAIDs, with bachelor’s degree (AOR = 6.92, 95% CI: 1.18–40.76) emerging as a significant factor.

#### 3.3.2. Assessing Contraindications Before Suggesting NSAID Use

In univariate analysis (Table 3), assessing contraindications was associated with Central region (COR = 2.36, 95% CI: 1.10–5.07), university facility (COR = 2.71, 95% CI: 1.52–4.84), Doctor of PT degree (COR = 18.20, 95% CI: 1.76–188.07), postgraduate degree (COR = 12.71, 95% CI: 1.51–107.21), and neurological (COR = 2.27, 95% CI: 1.12–4.60) and sports (COR = 2.40, 95% CI: 1.08–5.35) specializations. Only Doctor of PT degree (AOR = 11.46, 95% CI: 11.01–130.08) remained significant in the multivariate model.

#### 3.3.3. Monitoring Side Effects of Long-Term NSAID Use

In terms of monitoring long-term side effects, univariate analysis (Table 4) showed significant associations with Central region (COR = 4.01, 95% CI: 1.52–10.58) and orthopedic (COR = 3.57, 95% CI: 1.42–8.97), neurological (COR = 3.39, 95% CI: 1.28–8.95) pediatric (COR = 6.55, 95% CI: 2.06–20.84), geriatric (COR = 5.95, 95% CI: 1.43–24.78), and sports specialists (COR = 5.65, 95% CI: 2.02–15.81) specializations. In the multivariate model, therapists with 6–10 years of experience were less likely to monitor (AOR = 0.27, 95% CI: 0.09–0.80), while orthopedic (AOR = 3.49, 95% CI: 1.24–9.84), pediatric (AOR = 7.39, 95% CI: 1.99–27.42), geriatric (AOR = 4.83, 95% CI: 1.03–22.73), and sports specialists (AOR = 5.65, 95% CI: 1.77–17.99) were significantly more likely to monitor side effects compared with general practitioners.

#### 3.3.4. Recording Discussions on Medications

Univariate analysis indicated that Central (COR = 3.17, 95% CI: 1.26–8.01) and Northern regions (COR = 3.17, 95% CI: 1.17–8.60), university facilities (COR = 3.34, 95% CI: 1.86–6.01), and pediatric specialization (COR = 2.88, 95% CI: 1.04–7.95) were associated with higher odds of recording medication discussions. In the multivariate model, Central region (AOR = 2.87, 95% CI: 1.06–7.77) and university facility (AOR = 2.41, 95% CI: 1.18–4.94) remained significant predictors (Table 5).

### 3.4. Attitudes Toward NSAID Usage

A significant proportion of participants believed that PTs should hypothetically be able to prescribe NSAIDs (n = 141; 38.0%), compared with those who disagreed (n = 100; 27.0%), while more than one third (n = 130; 35.0%) were uncertain (*χ*^2^ = 7.28; *p* = 0.026). At the same time, most participants reported that their current knowledge was insufficient to advise patients on the safe use of NSAIDs (n = 230; 62.0%), and a further 26.1% (n = 97) were unsure (*χ*^2^ = 148.50; *p* < 0.001).

**Figure 4:**
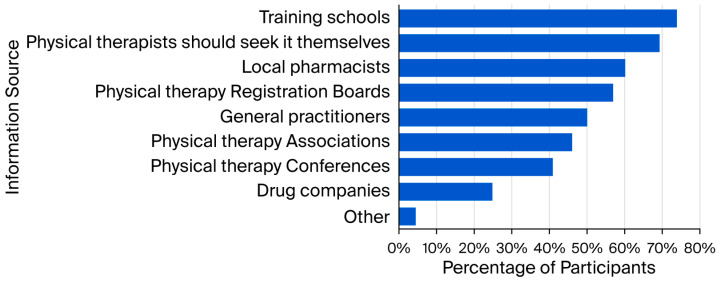
Perceived responsibility for providing NSAID information to physical therapists (n = 371; multiple responses).

Regarding responsibility for providing NSAID-related information to PTs, the majority identified training schools (n = 274; 73.9%), followed by self-directed learning (n = 257; 69.3%), local pharmacists (n = 223; 60.1%), registration boards (n = 211; 56.9%), general practitioners (n = 186; 50.1%), professional associations (n = 171; 46.1%), and conferences (n = 152; 41.0%). Fewer participants considered drug companies (n = 92; 24.8%) or other sources (n = 17; 4.6%) as responsible (Figure 4).

Of the 371 participants, 75 (20.2%) responded to the open-ended question on attitudes toward NSAIDs. Among these, 46.7% expressed supportive views, emphasizing their effectiveness in reducing pain and inflammation as adjuncts to physical therapy. Representative comments included:

“*I worked on myself to build up knowledge about it, thus I feel confident to advise patients*”.

In contrast, 17.3% were opposed, citing safety issues, scope-of-practice concerns, and need for further training. Examples included the following:

“*PTs should be able to prescribe NSAIDs, but only after sufficient training and passing a licensing exam*”.

The remaining 36.0% provided neutral or uncertain responses, reflecting a lack of knowledge and a tendency to rely on referral. Typical statements included:

“*I believe I am lacking information about these medications, which impacts my confidence. I often refer patients to doctors or pharmacists*”.

Overall, while nearly half of the responding PTs expressed positive views toward NSAID use as part of physical therapy care, a substantial number emphasized regulatory limitations, safety concerns, or the need for additional education before wider adoption in practice.

## 4. Discussion

To the best of the authors’ knowledge, this is the first nationwide study to comprehensively examine the practices and professional attitudes of PTs in Saudi Arabia regarding NSAID use in musculoskeletal care. The findings demonstrate that, although PTs in Saudi Arabia do not have prescribing authority, they are frequently involved in discussing, educating, and advising patients about NSAIDs as part of routine clinical practice. This highlights PTs’ expanding role as primary contact practitioners and their growing influence on patients’ medication-related decisions. However, the results also reveal important gaps in safety practices, documentation, and pharmacological knowledge that warrant critical attention. These insights also have broader relevance for countries where PTs function as first-contact practitioners.

More than half of the participating PTs reported routinely discussing NSAID use with patients, predominantly over-the-counter topical and oral formulations. This pattern is consistent with international evidence showing that PTs commonly discuss NSAIDs in additions to manual therapy and exercise, particularly in the management of acute and chronic musculoskeletal pain [9,21,22]. Despite this involvement, substantial safety concerns were identified. Less than half of PTs reported screening patients for contraindications, and fewer than one third monitored patients using NSAIDs in the long term. Furthermore, documentation of medication-related discussions was uncommon. These findings are concerning given the well-established gastrointestinal, renal, and cardiovascular risks associated with NSAIDs, particularly with prolonged use or in vulnerable populations [23]. In addition to systemic safety concerns, experimental evidence suggests that NSAIDs may influence biological processes related to tissue healing. For example, animal studies have demonstrated that selective COX-2 inhibitors can impede bone and tendon-to-bone healing and reduce prostaglandin-mediated signaling essential for tissue repair [24]. Furthermore, basic science research indicates that NSAIDs may negatively affect cellular proliferation and collagen synthesis in soft tissues, which are critical for musculoskeletal recovery [25]. While the current study did not explicitly measure PTs’ knowledge of these biological healing mechanisms, the observed gaps in monitoring suggest a potential lack of awareness regarding how NSAIDs influence the physiological stages of rehabilitation beyond simple pain modulation. The limited monitoring and documentation observed suggest that NSAID-related discussions may often be informal, inconsistently structured, and insufficiently integrated into clinical records.

While most PTs reported providing information about indications, fewer addressed drug interactions, precautions, side effects, or dosage. This selective counseling may reflect partial pharmacological knowledge or uncertainty about professional boundaries. Similar gaps have been reported internationally, where PTs reported low confidence in advising on drug interactions and contraindications [26,27]. The absence of formal pharmacology training in many physical therapy programs may contribute to this gap, limiting PTs’ ability to provide comprehensive patient education [15,28].

The likelihood of discussing NSAID use was higher among male PTs and those with greater clinical experience, which aligns with literature linking experience to increased confidence and autonomy in clinical decision-making [29]. PTs with orthopedic, neurological, and sports specializations were more likely to discuss NSAID use, likely reflecting greater exposure to pain-intensive conditions where adjunctive symptom management is common [18].

Notably, PTs working in university-affiliated facilities demonstrated better practices in assessing contraindications and recording medication discussions. This finding emphasize the role of academic settings in promoting evidence-based clinical practice [19].

Monitoring of long-term NSAID use was more common among pediatric, geriatric, and sports specialists, which may indicate heightened awareness of medication risks in vulnerable populations [30]. However, the overall low monitoring rates suggest that structured follow-up mechanisms and interprofessional collaboration remain insufficient across practice settings.

Attitudinal findings reveal considerable uncertainty within the physical therapy profession. Although over one third of participants supported the inclusion of clinical guidance on NSAIDs as part of physical therapy care, the majority reported insufficient knowledge to provide safe advice. This mismatch between clinical involvement and perceived competence raises important concerns about patient safety and professional accountability [31].

Qualitative responses (20%) further illustrated this tension. Supportive PTs described confidence gained through self-directed learning and collaboration with physicians or pharmacists, while others expressed reluctance due to safety concerns and scope-of-practice limitations. Neutral responses reflected uncertainty and reliance on referral, highlighting variability in confidence and preparedness across practitioners. These findings are similar to those reported from the United Kingdom, where early implementation of prescribing rights for PTs was met with similar uncertainty until structured competency frameworks were introduced [29]. Saudi PTs appear to have similar goals, but they do not have the systematic framework needed to carry them out safely.

Participants predominantly attributed responsibility for NSAID-related education to training institutions and self-learning, emphasizing gaps in structured continuing professional development. International experience suggests that embedding pharmacological modules into undergraduate and Continuing Professional Development. (CPD) curricula enhances PTs’ readiness to manage pain collaboratively [18,32,33].

### 4.1. Clinical Implications

- Undergraduate and postgraduate curricula could benefit from greater emphasis on pharmacovigilance, particularly regarding the identification of adverse effects and contraindications screening. Developing structured workshops or certification programs may further support PTs in providing evidence-based medication guidance.
- Healthcare facilities might consider establishing formal framework to guide NSAID-related discussions within physical therapy departments. Standardizing documentation and education protocols could help ensure that patient guidance is provided safely and consistently across all practice settings.
- There is an opportunity to enhance communication between PTs, pharmacists, and physicians to better coordinate pain management. By identifying risks in patients who self-medicate with over-the-counter (OTC) NSAIDs, PTs can potentially serve as a vital link in the interprofessional team, facilitating safer patient outcomes through formal referral pathways.

### 4.2. Strength and Limitations

This study represents the first nationwide assessment of Saudi PTs’ practices and attitudes toward NSAID use, adding important knowledge to the regional literature. The inclusion of a large, geographically diverse sample from various regions enhances the representativeness of the results, while comprehensive assessment of multiple NSAID-related behaviors—including discussing indications, screening for contraindication, monitoring side effects, and documenting conversation—provides a comprehensive understanding of clinical practices.

Nonetheless, several limitations should be acknowledged. The cross-sectional design precludes causal relationships between predictors and behaviors. Although the study achieved nationwide reach, the use of snowball sampling via social media may have introduced selection bias toward digitally active therapists, potentially limiting the findings’ generalizability to the broader Saudi PT workforce. Although the questionnaire was expert-reviewed and pilot-tested, the absence of formal psychometric evaluation may affect its measurement robustness. In addition, the instrument primarily focused on clinical practices and safety-related behaviors and did not comprehensively assess physiotherapists’ knowledge of NSAIDs’ biological effects on tissue healing, which may limit the interpretation of rehabilitation implications. Furthermore, the low response rate to the open-ended question suggests that the qualitative findings should be interpreted with caution, as they may not fully capture the diverse perspectives of the entire sample. Finally, reliance on self-reported data may have introduced recall or social desirability bias. Therefore, future studies may use objective clinical assessment or intervention-based designs to validate self-reported practices and assess the impact of targeted pharmacology education on PTs’ competence and confidence.

## 5. Conclusions

PTs in Saudi Arabia take an active role in NSAID-related guidance without any institutional support or training. While many PTs express positive attitudes towards integrating medication knowledge into practice, there are significant safety and ethical concerns due to gaps in knowledge, regulations, and documentation. To address these gaps and provide evidence-based, patient-centered care for musculoskeletal pain management, coordinated efforts in education, policy making, and interprofessional collaboration are needed.

## Figures and Tables

**Table 1:** Participant characteristics (n = 371).

Characteristics		n	%
Sex	Female	177	47.7
	Male	194	52.3
Geographical region in Saudi Arabia	Central	80	21.6
	Western	137	36.9
	Eastern	62	16.7
	Northern	48	12.9
	Southern	44	11.9
Working facility	Public	165	44.5
	Private	134	36.1
	University	72	19.4
Working experience (years)	Less than 1 year	37	10.0
	1–5 years	136	36.7
	6–10 years	96	25.9
	11 years or more	102	27.5
Degree	Diploma	8	2.2
	Bachelor	238	64.2
	Doctor of Physical Therapy	18	4.9
	Postgraduate	107	28.8
Specialization	Orthopedic	140	37.7
	Neurological	83	22.4
	Pediatric	25	6.7
	Geriatric	12	3.2
	Sports	47	12.7
	Other *	8	2.2
	General	56	15.1
Age mean (years) ± SD		33.3 ± 6.67	

Abbreviations: n = number; SD, standard deviation. * Other specializations included biomechanics, cardiopulmonary, pain management, and basic sciences.

**Table 2:** Logistic regression analysis of factors associated with physical therapists discussing NSAIDs with patients (n = 371).

Characteristics	Frequency		Univariate		Multivariate		VIF
	No	Yes	COR (95% CI)	*p*-Value	AOR (95% CI)	*p*-Value	
Sex							1.01
Female	88	89	1	-	1	-	
Male	64	130	2.01 (1.32–3.06)	0.001 *	1.72 (1.09–2.73)	0.021 *	
Geographical region in Saudi Arabia							1.01
Central	45	35	0.59 (0.28–1.24)	0.165	0.53 (0.24–1.21)	0.131	
Western	38	99	1.98 (0.98–4.00)	0.057	1.58 (0.73–3.42)	0.242	
Eastern	25	37	1.12 (0.51–2.46)	0.769	0.89 (0.38–2.07)	0.783	
Northern	25	23	0.70 (0.31–1.59)	0.394	0.67 (0.27–1.62)	0.373	
Southern	19	25	1	-	1	-	
Working facility							1.20
Public	69	96	1	-	1	-	
Private	56	78	1.00 (0.63–1.59)	0.996	1.43 (0.81–2.53)	0.213	
University	27	45	1.20 (0.68–2.12)	0.534	1.11 (0.54–2.26)	0.776	
Working experience (years)							1.54
Less than 1 year	24	13	1	-	1	-	
1–5 years	56	80	2.64 (1.24–5.62)	0.012 *	2.41 (1.04–5.60)	0.041 *	
6–10 years	38	58	2.82 (1.28–6.20)	0.010 *	2.62 (0.99–6.90)	0.052	
11 years or more	34	68	3.69 (1.67–8.14)	0.001 *	3.77 (1.31–10.86)	0.014 *	
Degree							1.70
Diploma	6	2	1	-	1	-	
Bachelor	98	140	4.29 (0.85–21.68)	0.078	6.92 (1.18–40.76)	0.032 *	
Doctor of Physical Therapy	10	8	2.40 (0.38–15.28)	0.354	5.55 (0.69–44.61)	0.107	
Postgraduate	38	69	5.45 (1.05–28.32)	0.044 *	5.67 (0.92–35.01)	0.062	
Specialization							1.09
Orthopedic	53	87	2.04 (1.09–3.81)	0.027 *	1.43 (0.70–2.92)	0.328	
Neurological	28	55	2.44 (1.21–4.89)	0.012 *	1.70 (0.76–3.79)	0.194	
Pediatric	15	10	0.83 (0.32–2.15)	0.697	0.84 (0.30–2.38)	0.744	
Geriatric	4	8	2.48 (0.67–9.20)	0.174	1.92 (0.47–7.76)	0.362	
Sports	16	31	2.40 (1.08–5.35)	0.032 *	1.56 (0.63–3.84)	0.338	
Other	5	3	0.74 (0.16–3.42)	0.704	0.66 (0.13–3.42)	0.616	
General	31	25	1	-	1	-	

Abbreviations: NSAIDs, Non-Steroidal Anti-Inflammatory Drugs; COR, crude odds ratio; CI, confidence interval; AOR, adjusted odds ratio; VIF, variance inflation factor. * Significant at α = 0.05.

**Table 3:** Logistic regression analysis of factors associated with physical therapists assessing contraindications before suggesting NSAID use (n = 371).

Characteristics	Frequency		Univariate		Multivariate		VIF
	No	Yes	COR (95% CI)	*p*-Value	AOR (95% CI)	*p*-Value	
Sex							1.01
Female	91	86	1	-	1	-	
Male	108	86	0.84 (0.56–1.27)	0.412	0.74 (0.47–1.18)	0.210	
Geographical region in Saudi Arabia							1.01
Central	36	44	2.36 (1.10–5.07)	0.027 *	1.69 (0.73–3.91)	0.216	
Western	78	59	1.46 (0.72–2.97)	0.294	1.28 (0.59–2.79)	0.536	
Eastern	34	28	1.59 (0.72–3.54)	0.254	1.39 (0.58–3.35)	0.459	
Northern	22	26	2.28 (0.98–5.31)	0.055	1.94 (0.78–4.83)	0.156	
Southern	29	15	1	-	1	-	
Working facility							1.20
Public	95	70	1	-	1	-	
Private	80	54	0.92 (0.58–1.46)	0.711	0.91 (0.52–1.59)	0.744	
University	24	48	2.71 (1.52–4.84)	0.001 *	1.60 (0.80–3.21)	0.185	
Working experience (years)							1.54
Less than 1 year	18	19	1	-	1	-	
1–5 years	84	52	0.59 (0.28–1.22)	0.153	0.65 (0.29–1.47)	0.299	
6–10 years	59	37	0.59 (0.28–1.28)	0.182	0.39 (0.15–1.00)	0.051	
11 years or more	38	64	1.60 (0.75–3.41)	0.228	0.76 (0.27–2.13)	0.608	
Degree							1.70
Diploma	7	1	1	-	1	-	
Bachelor	149	89	4.18 (0.51–34.55)	0.184	3.72 (0.41–33.57)	0.242	
Doctor of Physical Therapy	5	13	18.20 (1.76–188.07)	0.015 *	11.46 (1.01–130.08)	0.049 *	
Postgraduate	38	69	12.71 (1.51–107.21)	0.019 *	9.00 (0.95–84.99)	0.055	
Specialization							1.09
Orthopedic	77	63	1.73 (0.90–3.32)	0.100	1.52 (0.72–3.20)	0.268	
Neurological	40	43	2.27 (1.12–4.60)	0.023 *	1.43 (0.63–3.24)	0.393	
Pediatric	13	12	1.95 (0.74–5.11)	0.175	1.53 (0.53–4.41)	0.433	
Geriatric	5	7	2.96 (0.82–10.60)	0.096	2.24 (0.56–8.96)	0.255	
Sports	22	25	2.40 (1.08–5.35)	0.032 *	2.13 (0.87–5.27)	0.100	
Other	4	4	2.11 (0.47–9.41)	0.327	1.16 (0.22–6.16)	0.863	
General	38	18	1	-	1	-	

Abbreviations: NSAIDs, Non-Steroidal Anti-Inflammatory Drugs; COR, crude odds ratio; CI, confidence interval; AOR, adjusted odds ratio; VIF, variance inflation factor. * Significant at α = 0.05.

**Table 4:** Logistic regression analysis of factors associated with physical therapists monitoring side effects of long-term NSAID use (n = 371).

Characteristics	Frequency		Univariate		Multivariate		VIF
	No	Yes	COR (95% CI)	*p*-Value	AOR (95% CI)	*p*-Value	
Sex							1.01
Female	128	49	1	-	1	-	
Male	134	60	1.17 (0.75–1.83)	0.493	1.11 (0.67–1.84)	0.690	
Geographical region in Saudi Arabia							1.01
Central	49	31	4.01 (1.52–10.58)	0.005 *	2.66 (0.92–7.67)	0.071	
Western	98	39	2.52 (0.99–6.44)	0.053	2.34 (0.85–6.49)	0.101	
Eastern	44	18	2.59 (0.93–7.19)	0.068	2.73 (0.89–8.40)	0.079	
Northern	33	15	2.88 (1.00–8.27)	0.050	2.85 (0.91–8.96)	0.072	
Southern	38	6	1	-	1	-	
Working facility							1.20
Public	116	49	1	-	1	-	
Private	99	35	0.84 (0.50–1.39)	0.494	0.85 (0.45–1.62)	0.629	
University	47	25	1.26 (0.70–2.27)	0.443	0.78 (0.37–1.62)	0.504	
Working experience (years)							1.54
Less than 1 year	23	14	1	-	1	-	
1–5 years	106	30	0.46 (0.21–1.01)	0.054	0.46 (0.18–1.16)	0.101	
6–10 years	74	22	0.49 (0.22–1.11)	0.086	0.27 (0.09–0.80)	0.018 *	
11 years or more	59	43	1.20 (0.55–2.59)	0.647	0.44 (0.14–1.37)	0.156	
Degree							1.70
Diploma	6	2	1	-	1	-	
Bachelor	187	51	0.82 (0.16–4.18)	0.809	0.63 (0.10–3.92)	0.624	
Doctor of Physical Therapy	6	12	6.00 (0.92–39.18)	0.061	3.85 (0.48–30.79)	0.203	
Postgraduate	63	44	2.10 (0.40–10.87)	0.378	1.89 (0.30–12.13)	0.500	
Specialization							1.09
Orthopedic	98	42	3.57 (1.42–8.97)	0.007 *	3.49 (1.24–9.84)	0.018 *	
Neurological	59	24	3.39 (1.28–8.95)	0.014 *	2.83 (0.93–8.64)	0.067	
Pediatric	14	11	6.55 (2.06–20.84)	0.001 *	7.39 (1.99–27.42)	0.003 *	
Geriatric	7	5	5.95 (1.43–24.78)	0.014 *	4.83 (1.03–22.73)	0.046 *	
Sports	28	19	5.65 (2.02–15.81)	0.001 *	5.65 (1.77–17.99)	0.003 *	
Other	6	2	2.78 (0.45–16.98)	0.269	2.60 (0.36–18.61)	0.340	
General	50	6	1	-	1	-	

Abbreviations: NSAIDs, Non-Steroidal Anti-Inflammatory Drugs; COR, crude odds ratio; CI, confidence interval; AOR, adjusted odds ratio; VIF, variance inflation factor. * Significant at α = 0.05.

**Table 5:** Logistic regression analysis of factors associated with physical therapists recording discussions on medications in clinical records (n = 371).

Characteristics	Frequency		Univariate		Multivariate		VIF
	No	Yes	COR (95% CI)	*p*-Value	AOR (95% CI)	*p*-Value	
Sex							1.01
Female	123	54	1	-	1	-	
Male	137	57	0.95 (0.61–1.48)	0.813	0.87 (0.53–1.44)	0.596	
Geographical region in Saudi Arabia							1.01
Central	50	30	3.17 (1.26–8.01)	0.015 *	2.87 (1.06–7.77)	0.038 *	
Western	100	37	1.96 (0.80–4.77)	0.140	1.96 (0.76–5.07)	0.166	
Eastern	43	19	2.34 (0.88–6.17)	0.087	2.22 (0.79–6.29)	0.132	
Northern	30	18	3.17 (1.17–8.60)	0.023 *	2.49 (0.86–7.21)	0.093	
Southern	37	7	1	-	1	-	
Working facility							1.20
Public	127	38	1	-	1	-	
Private	97	37	1.27 (0.75–2.15)	0.364	1.31 (0.70–2.44)	0.393	
University	36	36	3.34 (1.86–6.01)	<0.001 *	2.41 (1.18–4.94)	0.016 *	
Working experience (years)							1.54
Less than 1 year	26	11	1	-	1	-	
1–5 years	101	35	0.82 (0.37–1.83)	0.626	0.97 (0.40–2.33)	0.937	
6–10 years	73	23	0.74 (0.32–1.74)	0.495	0.59 (0.21–1.65)	0.314	
11 years or more	60	42	1.65 (0.74–3.71)	0.222	0.90 (0.30–2.67)	0.847	
Degree							1.70
Diploma	7	1	1	-	1	-	
Bachelor	183	55	2.10 (0.25–17.47)	0.491	1.54 (0.17–13.93)	0.700	
Doctor of Physical Therapy	12	6	3.50 (0.35–35.37)	0.288	2.08 (0.18–23.67)	0.554	
Postgraduate	58	49	5.91 (0.70–49.74)	0.102	4.11 (0.44–38.55)	0.216	
Specialization							1.09
Orthopedic	95	45	1.74 (0.84–3.60)	0.138	1.31 (0.58–2.98)	0.518	
Neurological	62	21	1.24 (0.55–2.79)	0.599	0.68 (0.27–1.74)	0.427	
Pediatric	14	11	2.88 (1.04–7.95)	0.041 *	2.52 (0.83–7.62)	0.101	
Geriatric	8	4	1.83 (0.47–7.14)	0.382	1.08 (0.25–4.68)	0.918	
Sports	32	15	1.72 (0.71–4.17)	0.230	1.34 (0.50–3.63)	0.561	
Other	5	3	2.20 (0.46–10.55)	0.324	0.84 (0.14–5.00)	0.852	
General	44	12	1	-	1	-	

Abbreviations: COR, crude odds ratio; CI, confidence interval; AOR, adjusted odds ratio; VIF, variance inflation factor. * Significant at α = 0.05.

## Data Availability

The data presented in this study are available on request from the corresponding author due to ethical reasons.

## Supplementary Materials

The following supporting information can be downloaded at: https://www.mdpi.com/article/10.3390/healthcare14050591/s1, File S1: Reporting checklist for cross sectional study; File S2: Evaluating the practice and attitude of Saudi Physiotherapists toward NSAIDs.

## Abbreviations

The following abbreviations are used in this manuscript:

NSAIDs	Non-Steroidal Anti-Inflammatory Drugs
PT	Physical Therapist
PTs	Physical Therapists
COR	crude odds ratio
CI	confidence interval
AOR	adjusted odds ratio
SD	standard deviation.
VIF	variance inflation factor
CPD	Continuing Professional Development
OTC	Over The Counter
