# Supplementary material for: Physical Therapists’ Practices and Attitudes Toward Non-Steroidal Anti-Inflammatory Drugs: A National Cross-Sectional Study
Source: Healthcare (Basel). 2026 Feb 27;14(5):591. doi: 10.3390/healthcare14050591 (PMC12984721; doi:10.3390/healthcare14050591)
Supplement: Supplementary file 1 [file healthcare-14-00591-s001.zip › supplementary file S2.pdf]

Evaluating the practice and attitude of Saudi Physiotherapists toward NSAIDs

Part-1: Consent

Please read the following information carefully.

- **What is the purpose of the study?**
  - This study aims to explore physiotherapists' Practice and attitudes, toward NSAIDs.

- **You are eligible to participate in this survey if you meet the following criteria:**

  - Physiotherapy certificate holder

- **Do I have to take part?**
  - Your participation in this study is voluntary and you can withdraw at any time.

- **Risks of being a participant**
  - There are no foreseeable risks of being a participant in an online survey. This survey will not ask you about any identity.

- **Benefits of being a participant**
  - You may not receive direct benefit from participating in this study, but we anticipate the study findings will help us to better understand the current practice and attitudes of physiotherapists toward NSAIDs.

- **Time expected to complete this survey**
  - 7-10 minutes

- **Privacy & confidentiality?**
  - Your privacy will be maintained at all times, and you will not be identified, your data will be securely stored. If you have any questions about this research, please contact Dr. Wadi S Alruqayb PharmD, MSc, PhD w.alruqayb@tu.edu.sa Dr. Samia Alamrani PT, MSc, PhD Salamrani@ut.edu.sa,

Consent to participate

Please place a check if you agree to participate in this survey:

☐ Yes

☐ No

Part-2: Demographic information

1. **Please select your gender.**
   - ☐ Male
   - ☐ Female

2. **What is your age?**
   - ☐ Please write in number ……….

3. **In which region of Saudi Arabia do you currently work?**
   - ☐ Central Region (e.g., Riyadh, Qassim …etc)
   - ☐ Eastern Region (e.g., Dammam, Khobar, Jubail …etc)
   - ☐ Western Region (e.g., Jeddah, Mecca, Medina …etc)
   - ☐ Northern Region (e.g., Tabuk, Al-Jawf, Arar …etc)
   - ☐ Southern Region (e.g., Abha, Jizan, Najran …etc)

4. **What type of hospital do you work in?**
   - ☐ Public hospital/ centers/ clinics
   - ☐ University hospital/centers/ clinics
   - ☐ Private hospital /centers/ clinics
   - ☐ Other, specify…………..

5. **Please enter the years of your clinical experience.**
   - ☐ Less than one year
   - ☐ 1 – 5 years
   - ☐ 6 – 10 years
   - ☐ 11 – 20 years
   - ☐ More than 20 years

مقيد |TU| Restricted

6. **What is your highest earned academic degree?**
   - ☐ Diploma
   - ☐ Bachelor's Degree
   - ☐ Doctor of Physical Therapy (DPT)
   - ☐ Master's Degree
   - ☐ Doctoral Degree

7. **What is your area of specialization in physical therapy?**
   - ☐ Orthopedic
   - ☐ Neurological
   - ☐ Pediatric
   - ☐ Geriatric
   - ☐ Sports
   - ☐ Other (please specify): ___________

8. **Did your entry-level physiotherapy program include education on non-steroidal anti-inflammatory drugs (NSAIDs)?**
   - ☐ Yes
   - ☐ No
   - ☐ I Don't recall

9. When did you last update your knowledge on NSAIDs?

- ☐ Never
- ☐ < 1 year
- ☐ 1-5 years
- ☐ 5-10 years
- ☐ > 10 years

10. Did you actively seek out this information?

- ☐ Yes
- ☐ No

11. How did you obtain this information? (Multiple selection is allowed)

- ☐ Via another physiotherapist
- ☐ Journal (academic) articles
- ☐ General medical practitioner
- ☐ Local Pharmacist
- ☐ Training schools (e.g. university)
- ☐ Online search engine(e.g. google)
- ☐ Other (please specifiy) ______________________________

Part-1: Current Practice Regarding NSAID Usage

مقيّد |TU| Restricte

12. Does your practice/institution have a policy regarding physiotherapists recommending NSAIDs to patients?

- ☐ Yes
- ☐ No
- ☐ I Don't know

13. If Yes – Do you follow this policy?

- ☐ Yes
- ☐ No

14. If No, or Don't know – Do you think such a policy would be beneficial?

- ☐ Yes
- ☐ No
- ☐ I Don't know

15. Do you commonly recommend NSAIDs to your patients?

- ☐ Yes
- ☐ No

16. If yes, do your recommendations on NSAIDs include? (Multiple selection is allowed)

- ☐ Over the counter oral medication
- ☐ Over the counter topical medication
- ☐ Prescription oral medication
- ☐ Prescription topical medication
- ☐ Refer to GP
- ☐ Refer to pharmacist

17. **How often do you recommend NSAIDs?**
☐ Daily
☐ Weekly
☐ Monthly
☐ Rarely
☐ Never

18. **What are the common indications for recommending NSAIDs in your practice? (Multiple selection is allowed)**
☐ Acute pain (e.g., musculoskeletal injuries)
☐ Chronic pain (e.g., osteoarthritis, rheumatoid arthritis)
☐ Fever
☐ Inflammation (e.g., tendinitis, bursitis)
☐ Dysmenorrhea (menstrual cramps)
☐ Prophylactic use (e.g., cardiovascular prevention with aspirin)
☐ Other (please specify): __________

19. **Do you assess patients for contraindications before recommending NSAIDs?**
☐ Yes
☐ No

20. **Do you monitor patients on long-term NSAIDs for side effects?**
☐ Yes
☐ No

مقيد |TU| Restricted

21. **How do you monitor patients on long-term NSAIDs for side effects? (Multiple selection is allowed)**
☐ Routine laboratory tests (e.g., renal function, liver enzymes)
☐ Regular blood pressure checks
☐ Patient-reported symptoms (e.g., gastrointestinal issues)
☐ Scheduled follow-up visits
☐ Other (please specify): ___________

22. **Do you record any discussions on medications in your clinical records?**
☐ Yes
☐ No

30. **Do your recommendations on the use of NSAIDs include?**

a) Warnings/advice
☐ Yes
☐ No

b) Indications
☐ Yes
☐ No

c) Precautions

- ☐ Yes
- ☐ No

d) Contraindications
- ☐ Yes
- ☐ No

e) Drug interactions
- ☐ Yes
- ☐ No

f) Side effects
- ☐ Yes
- ☐ No

g) Dosage
- ☐ Yes
- ☐ No

Part-2: ATTITUDES TO NSAIDs

23. Do you believe that physiotherapists should be able to prescribe NSAIDs?
- ☐ Yes
- ☐ No
- ☐ I Don't know

24. Do you believe that your current knowledge of NSAIDs is sufficient to allow you to advise patients on safe use?
- ☐ Yes
- ☐ No
- ☐ I Don't know

مقيد | TU | Restricted

25. Whose responsibility do you think it is, to provide information on NSAIDs to physiotherapists (Multiple selection is allowed)

- ☐ Physiotherapists should seek it themselves
- ☐ Drug companies
- ☐ Physiotherapy Registration Boards
- ☐ Physiotherapy Association
- ☐ Local pharmacists
- ☐ General practitioners
- ☐ Training schools (e.g. School of Physiotherapy/Pharmacy in a university)
- ☐ Physiotherapy conferences
- ☐ Other (please describe)

26. Could you briefly summarize your attitude toward the role of NSAIDs in physiotherapy practice?

……………………………………………………………………………………
……………………………………………………………………………………
……………………………………………………………………………………
